# Folic Acid Ameliorates Neuronal Ferroptosis in Aging by Up-Regulating SLC7A11-GSH-GPX_4_ Antioxidant Pathway and Increasing Cystine Levels

**DOI:** 10.3390/ijms26146669

**Published:** 2025-07-11

**Authors:** Yue Wang, Jingwen Zhang, Zehao Wang, Qinghan Ren, Zhenshu Li, Guowei Huang, Wen Li

**Affiliations:** 1 Department of Nutrition and Food Science, School of Public Health, Tianjin Medical University, Tianjin 300070, China; wangyue9923@tmu.edu.cn (Y.W.); zhangjw02@tmu.edu.cn (J.Z.); wangzehao@tmu.edu.cn (Z.W.); renqinghan0223@tmu.edu.cn (Q.R.); lizhenshu@tmu.edu.cn (Z.L.); huangguowei@tmu.edu.cn (G.H.); 2 Tianjin Key Laboratory of Environment, Nutrition and Public Health, Tianjin 300070, China; 3 Key Laboratory of Prevention and Control of Major Diseases in the Population, Ministry of Education, School of Public Health, Tianjin Medical University, Tianjin 300070, China

**Keywords:** folic acid, ferroptosis, cystine, aging, SLC7A11, neuroprotection

## Abstract

Age-related neurodegeneration is characterized by oxidative stress and iron-dependent cell death, yet the neuroprotective mechanisms of folic acid in modulating ferroptosis remain unclear. This study systematically investigated the role of folic acid in inhibiting ferroptosis and attenuating neuronal damage in aging, with a focus on the solute carrier family 7 member 11 (SLC7A11)-glutathione (GSH)-glutathione peroxidase 4 (GPX_4_) antioxidant pathway, using aged rats supplemented with folic acid (<0.1, 2.0, and 4.0 mg/kg·diet) for 22 months, with young adult rats as controls. Brain iron accumulation and ferroptosis-related proteins (SLC7A11, GPX_4_, Ferritin heavy chain 1 (FTH1)) were evaluated. In vitro, HT-22 hippocampal neuronal cells were pre-treated with folic acid (0, 10, 20 μmol/L) for 72 h before combining with Erastin (10 μmol/L)-induced ferroptosis for an additional 24 h. Intracellular Fe^2+^, lipid peroxidation (LPO), malondialdehyde (MDA), reactive oxygen species (ROS), along with cystine, GSH, and ferroptosis-related protein levels were quantified. Stable sh-*SLC7A11* knockdown and control (sh-NC) cell lines were used to validate the dependency of folic acid’s protective effects on SLC7A11 expression. Folic acid supplementation in aged rats dose-dependently reduced aging-related brain iron accumulation and enhanced the expression of SLC7A11, GPX_4_, and FTH1. In Erastin-induced HT-22 cells, folic acid significantly mitigated ferroptosis hallmarks. Mechanistically, folic acid increased extracellular cystine uptake and intracellular GSH synthesis, thereby activating the SLC7A11-GSH-GPX_4_ antioxidant pathway. Notably, molecular docking technique suggested that compared to GPX_4_, folic acid stabilized SLC7A11’s active conformation. sh-*SLC7A11* knockdown completely abolished folic acid-mediated protection against ferroptosis, as evidenced by restored loss of cystine, GSH and GPX_4_ production. This study innovatively emphasized the critical role of folic acid supplementation in inhibiting ferroptosis by up-regulating the SLC7A11-GSH-GPX_4_ antioxidant pathway, primarily through enhancing cystine availability and SLC7A11 expression. These findings established folic acid as a potential dietary intervention for aging-related neurodegenerative diseases characterized by neuronal ferroptosis, providing preclinical evidence for folic acid based neuroprotection.

## 1. Introduction

The global population aging has become one of the most significant and accelerating demographic trends of our time, with far-reaching social, economic, and public health challenges [1,2]. Aging is accompanied by progressive oxidative stress and iron metabolic dysregulation, which converge to drive ferroptosis, a form of iron-dependent lipid peroxidation (LPO)-mediated cell death [3,4]. Brain neurons have cell membranes rich in polyunsaturated fatty acids, which make them vulnerable to ferroptosis-related attacks such as reactive oxygen species (ROS) [5]. This pathological process is implicated in neuronal damage and neurodegenerative diseases [6]. While ferroptosis has emerged as a key therapeutic target for aging-related brain disorders, the role of folic acid in modulating this pathway remains underexplored.

Folic acid is a B vitamin that plays several important roles in neuroprotection. Folic acid acts as a methyl donor and has antioxidant properties. Folic acid can cross the blood–brain barrier and enter the brain tissue. In in vivo and in vitro experiments, folic acid supplementation inhibited apoptosis in neuronal cells by attenuating oxidative stress and preventing telomere attrition [7,8]. Folic acid deficiency causes hyperhomocysteinemia (HHcy) [9], and high levels of Hcy induce oxidative stress damage to neurons [10]. Under normal physiological conditions, Hcy is oxidized and converted to cysteine that participates in substrate of glutathione peroxidase 4 (GPX4) called glutathione (GSH) synthesis, thereby increasing the activity of this antioxidant enzyme resists oxidative stress and ferroptosis [11]. Cysteine is highly susceptible to oxidation to cystine, which is taken up into the cell by the cystine/glutamate transporter, solute carrier family 7 member 11 (SLC7A11). However, there is no direct evidence that folic acid can affect ferroptosis by modulating SLC7A11-GSH-GPX4 antioxidant pathway and cystine levels, thereby ameliorating oxidative damage to neurons in the aging brain. Exploring the relationship between folic acid and ferroptosis provides a novel mechanistic basis for studies to attenuate aging-induced neuronal damage and ameliorate neurodegenerative lesions.

The aim of this study is to use naturally aged rats and ferroptosis models of mouse HT-22 neuronal cells to reveal how folic acid affects ferroptosis mechanism to exert neuroprotective effects. We hypothesized that folic acid inhibited ferroptosis by up-regulating SLC7A11-GSH-GPX_4_ pathway and improving neuronal intracellular cystine levels, thereby attenuating aging-related neuronal damage.

## 2. Results

### 2.1. Folic Acid Supplementation Inhibited Age-Related Brain Iron Deposition and Increased Cystine Levels in Rats

**Figure 1:**
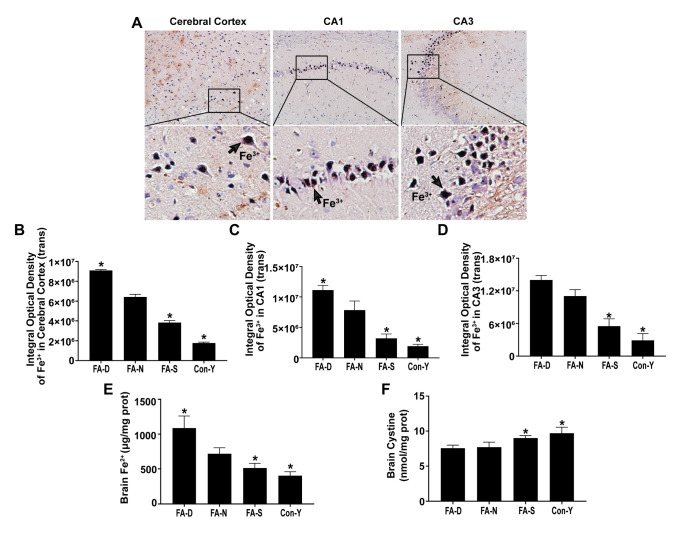
Folic acid supplementation inhibited age-related brain iron deposition and increased cystine levels in rats. Three-month-old male rats were randomly divided into three intervention groups (n = 30)—folic acid-deficient diet group (FA-D), folic acid-normal diet group (FA-N), folic acid-supplemented diet group (FA-S)—containing folic acid <0.1, 2.0, and 4.0 mg/kg diet, respectively. In addition, a group of four-month-old male SD rats served as the young control (Con-Y) group (n = 10). (**A**) Representative micrographs showed positive DAB-enhanced Perls’ staining (indicated by brown dots) for Fe^3+^ within the cerebral cortex and the CA1 and CA3 regions of the hippocampus in rats, with arrows highlighting areas of Fe^3+^ deposition (n = 3 rats/group). Scale bar = 50 μm. (**B**–**D**) Quantitative analysis of Fe^3+^ levels in the cerebral cortex and the CA1 and CA3 regions of the hippocampus (n = 3 rats/group). Panel (**B**) depicts cerebral cortex, panel (**C**) depicts CA1, and panel (**D**) depicts CA3. (**E**) Fe^2+^ concentrations measured in brain tissue of rats (n = 6 rats/group). (**F**) Cystine levels measured in brain tissue of rats (n = 6 rats/group). Data are presented as mean ± SD. Statistical significance was indicated as * *p* < 0.05 compared to the FA-N group.

A comparison between aged rats (FA-N group) and young rats (Con-Y group) revealed a significant increase in Fe^3+^ levels in the cerebral cortex and the hippocampus CA1 and CA3 regions (*p* < 0.05, Figure 1A–D). Additionally, there was a notable rise in Fe^2+^ levels in brain tissue of aged rats (*p* < 0.05, Figure 1E).

**Figure A1:**
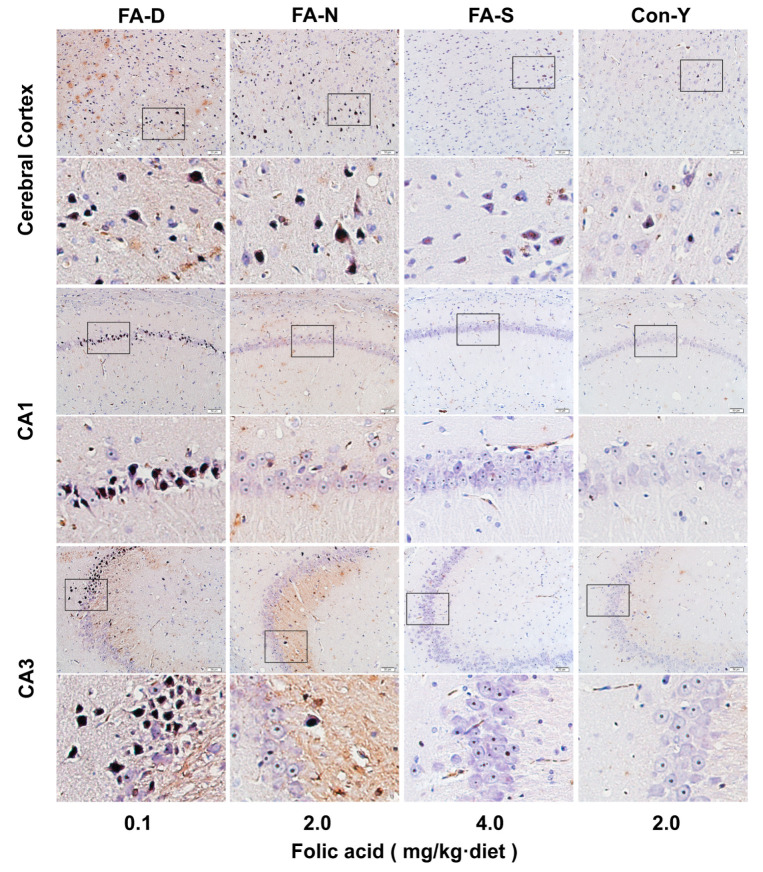
Folic acid supplementation inhibited age-related brain iron deposition in aging rats. Representative micrographs showed positive DAB-enhanced Perls’ staining (indicated by brown dots) for Fe^3+^ deposition within the cerebral cortex and the CA1 and CA3 regions of the hippocampus in rats (n = 3 rats/group). Below each image with a rectangular box was a zoomed-in image of the area within the rectangular box. Scale bar = 50 μm.

Importantly, brain iron deposition decreased with folic acid supplementation. Specifically, levels of Fe^3+^ in the cerebral cortex and the hippocampus CA1 and CA3 regions (*p* < 0.05, Figure 1A–D), as well as Fe^2+^ in brain tissue (*p* < 0.05, Figure 1E), progressively decreased as the dosage of folic acid increased. The lowest iron levels were found in the FA-S group, while the highest were noted in the FA-D group. All representative micrographs showed positive DAB-enhanced Perls’ staining (indicated by brown dots) for Fe^3+^ deposition within the cerebral cortex, and the CA1 and CA3 regions of the hippocampus in rats are shown in Figure A1.

However, cystine levels in brain tissue of aged rats were significantly lower than those of the young ones (*p* < 0.05, Figure 1F). Among aged rats, brain cystine levels showed a resumption of upward trend with folic acid supplementation that was the highest in FA-S group (*p* < 0.05, Figure 1F).

These findings indicated the occurrence of age-related brain iron deposition in rats, with the most significant accumulation linked to folic acid deficiency. Brain tissue cystine levels were also age-related, and folic acid deficiency can lead to cystine decreased. Furthermore, folic acid supplementation effectively reduced age-related brain iron deposition and increased cystine levels.

### 2.2. Folic Acid Supplementation Counteracted the Reduction in the Expression of Ferroptosis-Inhibitory Proteins in the Brains of Aged Rats

**Figure 2:**
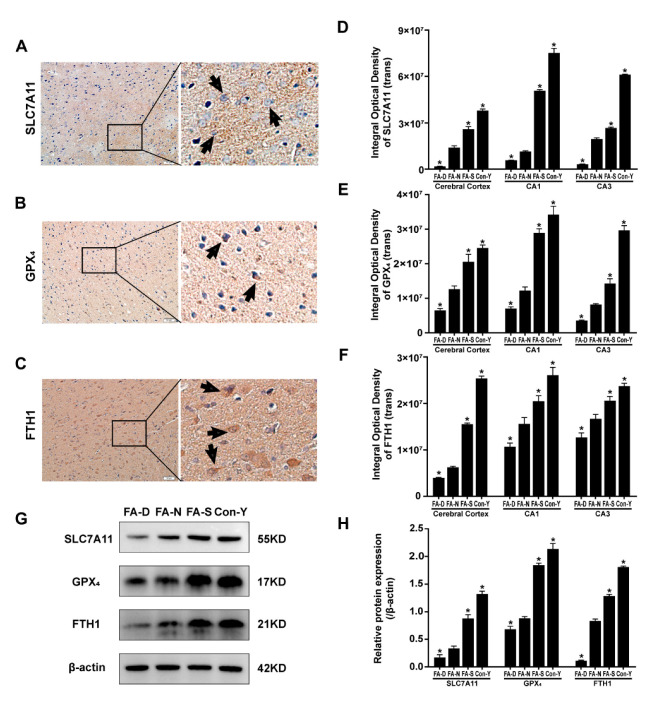
Folic acid supplementation counteracted the reduction in the expression of ferroptosis-inhibitory proteins in the brains of aged rats. Rats were assigned to treatment groups as described in Figure 1. (**A**–**C**) Representative micrographs from immunohistochemical staining of ferroptosis-inhibitory proteins were presented, with arrows indicating positive staining. Scale bar = 50 μm. Panel (**A**) depicts SLC7A11, panel (**B**) depicts GPX_4_, and panel (**C**) depicts FTH1. (**D**–**F**) Quantitative analysis of the expression levels of these proteins in the cerebral cortex and the CA1 and CA3 regions of the hippocampus in rats (n = 3 rats/group). Panel (**D**) depicts SLC7A11, panel (**E**) depicts GPX_4_, and panel (**F**) depicts FTH1. (**G**) Representative Western blots showed SLC7A11, GPX_4_, FTH1, and β-actin levels in brain tissue (n = 6 rats/group). (**H**) SLC7A11, GPX_4_, and FTH1 protein expression levels are normalized to β-actin (n = 6 rats/group). Data are presented as mean ± SD. Statistical significance is indicated as * *p* < 0.05 compared to the FA-N group.

**Figure A2:**
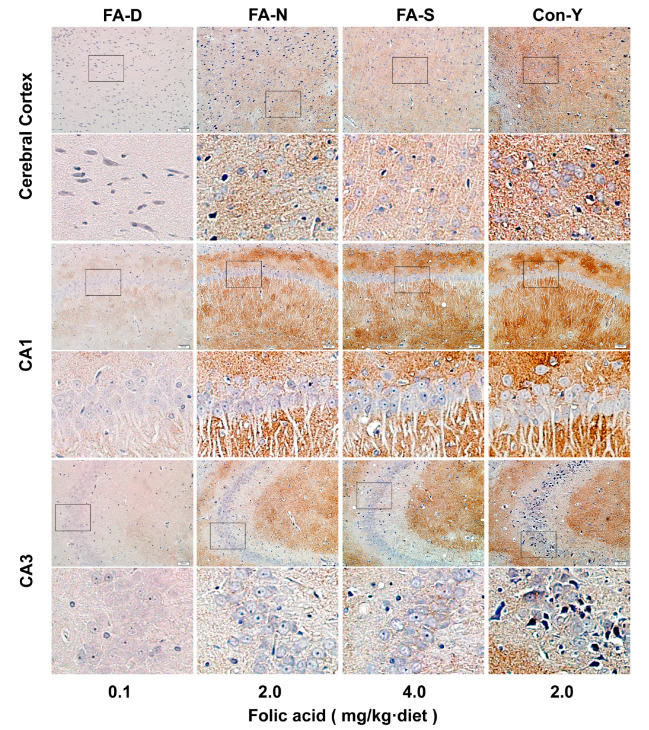
Folic acid supplementation counteracted the reduction in the expression of SLC7A11 proteins in the brains of aged rats. Representative micrographs from immunohistochemical staining of SLC7A11 proteins within the cerebral cortex and the CA1 and CA3 regions of the hippocampus in rats. (n = 3 rats/group). Below each image with a rectangular box was a zoomed-in image of the area within the rectangular box. Scale bar = 50 μm.

**Figure A3:**
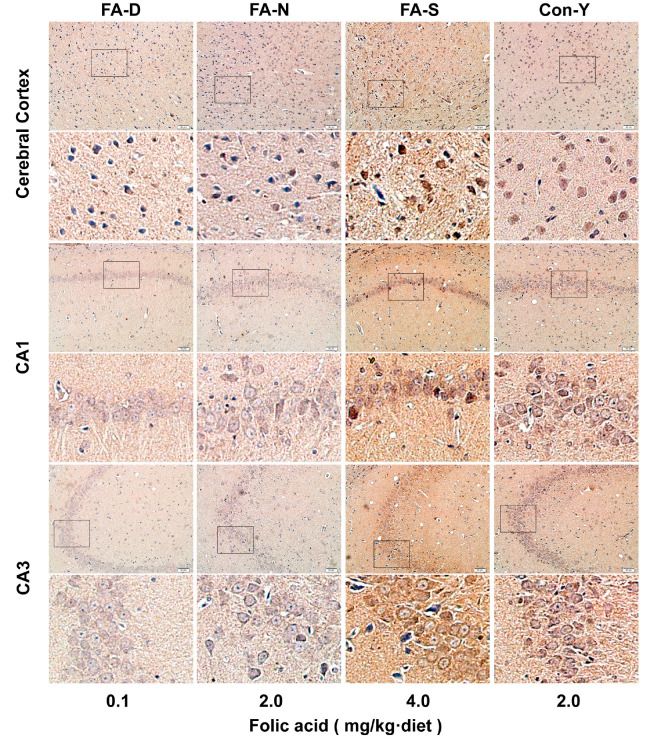
Folic acid supplementation counteracted the reduction in the expression of GPX_4_ proteins in the brains of aged rats. Representative micrographs from immunohistochemical staining of GPX_4_ proteins within the cerebral cortex and the CA1 and CA3 regions of the hippocampus in rats. (n = 3 rats/group). Below each image with a rectangular box was a zoomed-in image of the area within the rectangular box. Scale bar = 50 μm.

**Figure A4:**
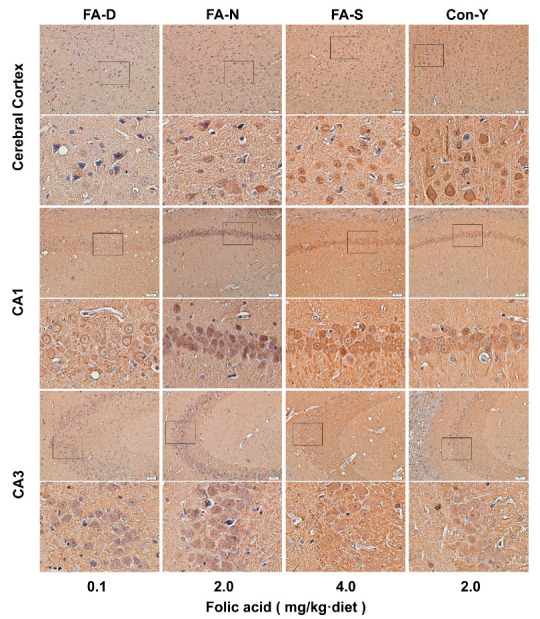
Folic acid supplementation counteracted the reduction in the expression of FTH1 proteins in the brains of aged rats. Representative micrographs from immunohistochemical staining of FTH1 proteins within the cerebral cortex and the CA1 and CA3 regions of the hippocampus in rats. (n = 3 rats/group). Below each image with a rectangular box was a zoomed-in image of the area within the rectangular box. Scale bar = 50 μm.

Immunohistochemical staining analysis revealed that aged rats (FA-N group) exhibited reduced protein levels of SLC7A11, GPX_4_, and Ferritin heavy chain 1 (FTH1) in the cerebral cortex, as well as in the hippocampus CA1 and CA3 regions, compared to young rats (Con-Y group). Increasing doses of folic acid effectively reversed this decline, with the highest protein levels observed in the FA-S group, followed by the FA-N group, while the lowest levels were observed in the FA-D group (*p* < 0.05, Figure 2A–F). All representative micrographs from immunohistochemical staining of SLC7A11, GPX_4_, and FTH1 proteins within the cerebral cortex and the CA1 and CA3 regions of the hippocampus in rats are shown in Figure A2, Figure A3 and Figure A4.

Further confirmation was provided by Western blot analysis, which indicated that aged rats (FA-N group) had lower levels of SLC7A11, GPX_4_, and FTH1 proteins in brain tissue compared to the young rats (Con-Y group). The expression levels of these ferroptosis inhibitory proteins increased significantly with folic acid supplementation, showing the highest levels in the FA-S group, intermediate in the FA-N group, and the lowest in the FA-D group (*p* < 0.05, Figure 2G,H).

These findings suggested that the expression of ferroptosis inhibitory proteins SLC7A11, GPX_4_, and FTH1 in the brains of rats declines with age, particularly under conditions of folic acid deficiency. Notably, folic acid supplementation effectively reversed this decline in the brain.

### 2.3. Folic Acid Supplementation Inhibited Ferroptosis in Erastin-Induced HT-22 Neuronal Cells

To investigate the mechanism by which folic acid regulates ferroptosis, we treated HT-22 cells in vitro with the ferroptosis inducer Erastin and examined the effects of folic acid supplementation.

**Figure 3:**
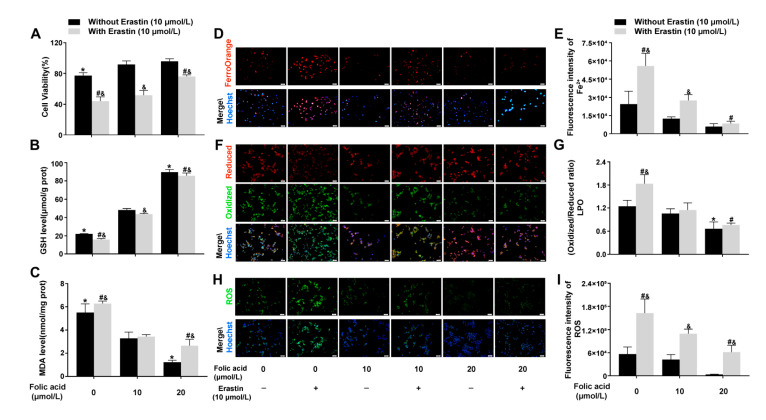
Folic acid supplementation inhibited ferroptosis in Erastin-induced HT-22 neuronal cells. HT-22 cells were randomly divided into six intervention groups: (1) folic acid-deficient without Erastin group (−0); (2) folic acid-deficient with Erastin group (+0); (3) folic acid-normal without Erastin group (−10); (4) folic acid-normal with Erastin group (+10); (5) folic acid-supplemented without Erastin group (−20); (6) folic acid-supplemented with Erastin group (+20). HT-22 cells were incubated for 72 h with folic acid (0, 10, 20 μmol/L) and then for 24 h with a combination of folic acid and either Erastin (10 μmol/L) (+) or vehicle(−). (**A**) Cell viability of HT-22 cells was assessed by CCK-8 assay (n = 5/group). (**B**) Quantitative analysis of GSH levels (n = 6/group). (**C**) Quantitative analysis of MDA levels (n = 6/group). (**D**,**E**) Representative micrographs and quantitative analysis of intracellular Fe^2+^ (n = 3/group). Scale bar = 50 μm. (**F**,**G**) Representative micrographs and quantitative analysis of intracellular LPO (n = 3/group). Scale bar = 50 μm. (**H**,**I**) Representative micrographs and quantitative analysis of intracellular ROS (n = 3/group). Scale bar = 50 μm. Data are expressed as mean ± SD. Statistical significance is indicated as * *p* < 0.05 compared with the folic acid-normal without Erastin group; # *p* < 0.05 compared with the folic acid-normal with Erastin group; and & *p* < 0.05 compared with the same folic acid concentration.

The CCK-8 assay revealed that folic acid exhibits neuroprotective effects in an Erastin-induced ferroptosis model involving HT-22 cells, where Erastin notably reduced cell viability. However, supplementation with folic acid mitigated the decrease in cell viability caused by ferroptosis. Specifically, compared to the 0 μmol/L folic acid (folic acid-deficient) group, which demonstrated a viability of 44.0% ± 5.6%, the 10 μmol/L and 20 μmol/L folic acid resulted in increased cell viability in the presence of Erastin (51.8% ± 6.2% and 76.1% ± 1.9%, respectively), with statistical significance (*p* < 0.05, Figure 3A). Furthermore, Erastin-induced ferroptosis led to a reduction in GSH levels and an increase in MDA levels; this phenomenon was exacerbated by folic acid deficiency but effectively inhibited by folic acid supplementation. Compared to the 10 μmol/L folic acid group, supplementation with 20 μmol/L folic acid resulted in elevated GSH levels and decreased MDA levels in the presence of Erastin (*p* < 0.05, Figure 3B,C). Cell fluorescence probe results indicated that Erastin-induced ferroptosis significantly increased Fe^2+^, LPO, and ROS levels. In comparison with the 10 μmol/L folic acid group, 20 μmol/L folic acid significantly reduced levels of Fe^2+^, LPO, and ROS in the presence of Erastin (*p* < 0.05, Figure 3D–I).

These results indicated that cell damage caused by ferroptosis was most severe in cases of folic acid deficiency. Folic acid offered protection to HT-22 neurons from ferroptosis by lowering levels of intracellular Fe^2+^, LPO, MDA, and ROS. Additionally, it promoted the production of GSH, boosted antioxidant capacity and inhibited lipid peroxidation.

### 2.4. Folic Acid Supplementation Counteracted the Reduction in the Expression of Ferroptosis-Inhibitory Proteins and Increased Cystine Levels in Erastin-Induced HT-22 Cells

**Figure 4:**
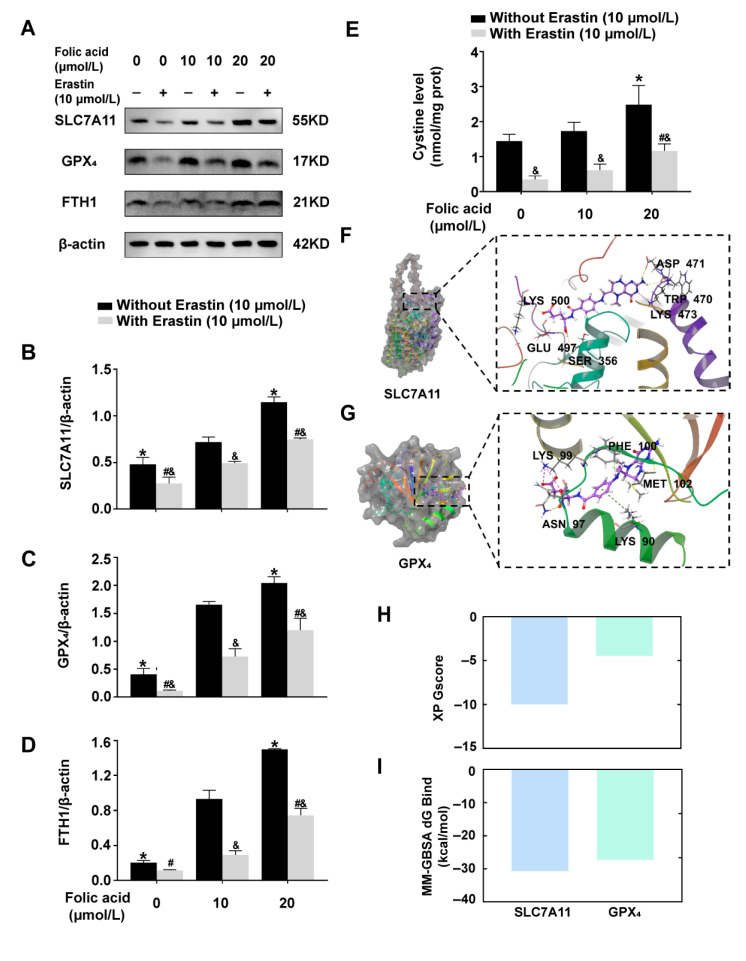
Folic acid supplementation counteracted the reduction in the expression of ferroptosis-inhibitory proteins in Erastin-induced HT-22 cells. Cells were assigned to treatment groups as described in Figure 3. (**A**) Representative Western blots of cells SLC7A11, GPX_4_, FTH1 and β-actin (n = 3/group). (**B**–**D**) The protein expression levels of SLC7A11, GPX_4_, FTH1 were normalized to β-actin (n = 3/group). Panel (**B**) depicts SLC7A11, panel (**C**) depicts GPX_4_, and panel (**D**) depicts FTH1. (**E**) Quantitative analysis of cystine levels (n= 6/group). (**F**) 3D molecular dock between 5-MTHF and SLC7A11 crystal structure (predicted by AlphaFold2). Hydrogen bonds (yellow dotted line), salt bridge (purple dotted line). (**G**) Three-dimensional molecular dock between 5-MTHF and GPX_4_ crystal structure (PDB ID: 5L71). Hydrogen bonds (yellow dotted line), salt bridge (purple dotted line), and π-π stacking interaction (green dotted line). (**H**) 5-MTHF docked to SLC7A11 (blue column)or GPX_4_ (green column) XP GScore analysis. (**I**) 5-MTHF docked to SLC7A11 (blue column) or GPX_4_ (green column) MM-GBSA dG Bind analysis. Data are expressed as mean ± SD. Statistical significance is indicated as * *p* < 0.05 compared with the folic acid-normal without Erastin group; # *p* < 0.05 compared with the folic acid-normal with Erastin group; and & *p* < 0.05 compared with the same folic acid concentration.

Western blot analysis indicated that Erastin-induced ferroptosis significantly diminished the expression of ferroptosis inhibitory proteins SLC7A11, GPX_4_, and FTH1 in HT-22 cells, particularly in the folic acid-deficiency group. In comparison to the folic acid 10 μmol/L group, the addition of 20 μmol/L folic acid led to an increase in the expression levels of SLC7A11, GPX_4_, and FTH1 proteins in the presence of Erastin (*p* < 0.05, Figure 4A–D). Consistent with the in vivo results, these findings suggested that folic acid mitigates ferroptosis in neuronal cells by enhancing the SLC7A11-GSH-GPX_4_ antioxidant pathway.

Cystine levels were all significantly reduced in the ferroptosis cells model, particularly in the folic acid-deficiency group. In comparison to the folic acid 10 μmol/L group, the cystine level of folic acid 20 μmol/L group was significantly improved (*p* < 0.05, Figure 4E).

**Figure A5:**
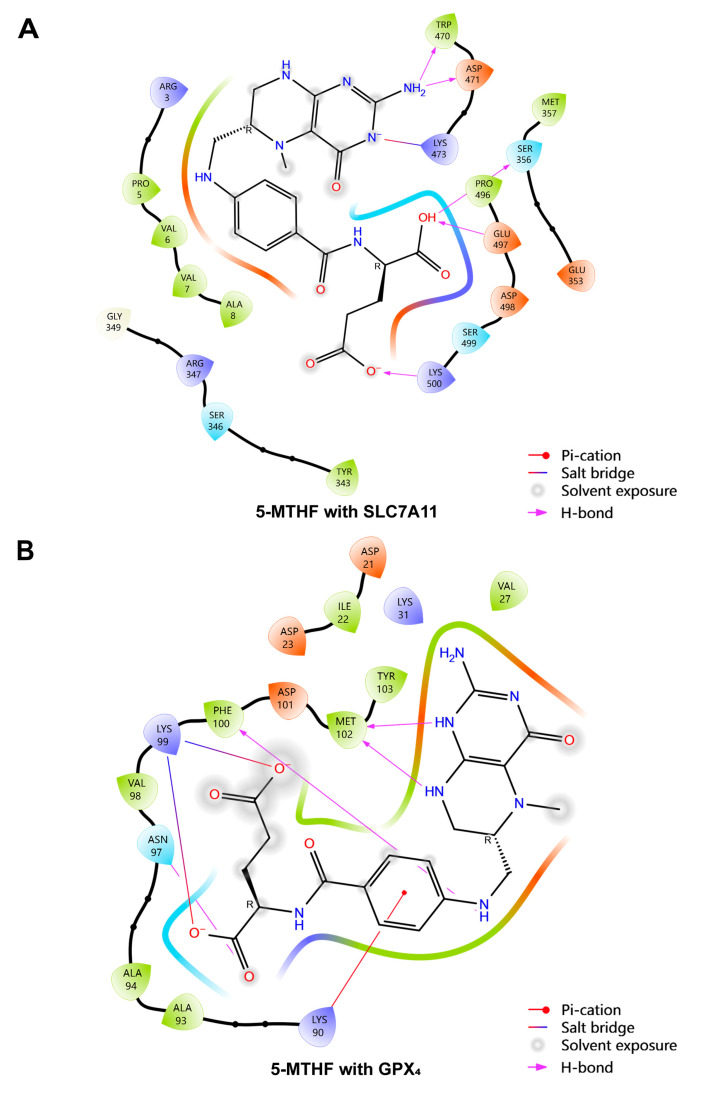
The 2D ligand–protein interaction diagram of the molecular docking pose among 5-MTHF, SLC7A11, and GPX_4_ crystal structures. (**A**) The 2D ligand–protein interaction diagram of the molecular docking pose between 5-MTHF and SLC7A11 crystal structure (predicted by AlphaFold2). (**B**) The 2D ligand–protein interaction diagram of the molecular docking pose between 5-MTHF and GPX_4_ crystal structure (PDB ID:5L71). 5-MTHF bound to the active pocket of SLC7A11 protein and formed a hydrogen bond with the residues LYS500, GLU497, SER356, ASP471, and TRP470 of SLC7A11 protein, and a hydrogen bond and a salt bridge with the residue LYS473. 5-MTHF bound to the active pocket surface of GPX4 protein, forming a hydrogen bond with protein residues PHE100 and ASN97, two hydrogen bonds with residue MET102, two salt bridges with residue LYS99, and a π-Cation bond with residue LYS90.

To investigate the potential inhibition of ferroptosis by folic acid, molecular docking studies evaluated the binding affinity of 5-methyltetrahydrofolate (5-MTHF) (the specific active form of folic acid in vivo) to both upstream SLC7A11 and downstream GPX_4_. The highest extra precision (XP) analysis indicated that 5-MTHF effectively bound to the active site of SLC7A11, forming hydrogen bonds with LYS500, GLU497, SER356, ASP471, and TRP470, as well as a hydrogen bond and salt bridge with LYS473 (Figure 4F). In the case of GPX_4_, 5-MTHF bound to the surface of the pocket, establishing hydrogen bonds with PHE100, ASN97, and two bonds with MET102, along with salt bridges involving LYS99 and a π-cation bond with LYS90 (Figure 4G). The 2D molecular docks between 5-MTHF and SLC7A11, as well as 5-MTHF and GPX_4_ are shown in Figure A5A,B. Both XP docking and molecular mechanics/generalized born surface area f binding free energy (MM-GBSA dG Bind) demonstrated stable binding of 5-MTHF to SLC7A11 (XP GScore: −9.989; MM-GBSA dG Bind: −30.73 kcal/mol) while revealing unstable binding to GPX_4_ (XP GScore: −4.475; MM-GBSA dG Bind: −27.24 kcal/mol) (Figure 4H,I).

These findings suggested that folic acid may serve as a potential regulator of SLC7A11, modulating its function through binding, subsequently influencing the SLC7A11-GSH-GPX_4_ antioxidant pathway and the associated indicators of ferroptosis. And folic acid supplementation reversed the decline in cystine in the ferroptosis cells model.

### 2.5. Folic Acid Inhibited Ferroptosis by Acting on SLC7A11 to Up-Regulate SLC7A11-GSH-GPX_4_ Pathway and Increase Cystine Levels

**Figure 5:**
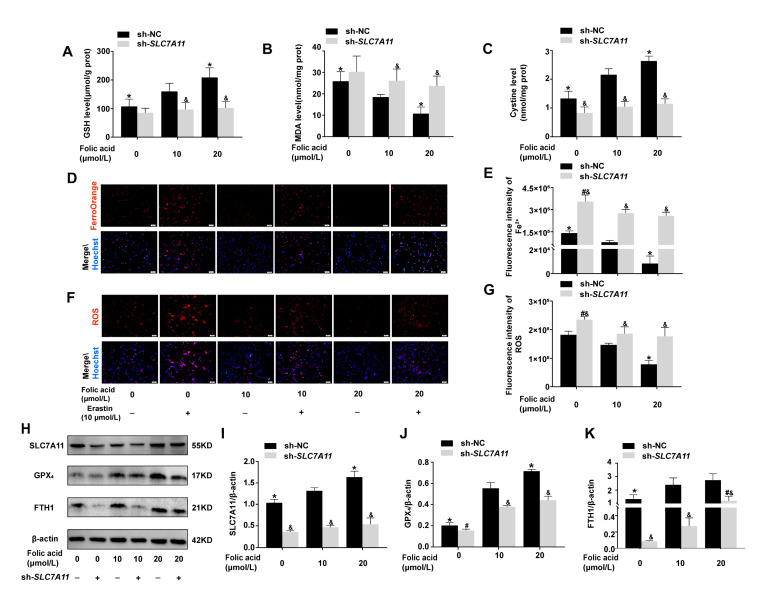
Folic acid inhibited ferroptosis by acting on SLC7A11 to up-regulate SLC7A11-GSH-GPX_4_ pathway and increase cystine levels. HT-22 cells were randomly divided into six intervention groups: (1) sh-NC folic acid-deficient group (N0); (2) sh-*SLC7A11* folic acid-deficient group (S0); (3) sh-NC folic acid-normal group (N10); (4) sh-*SLC7A11* folic acid-normal group (S10); (5) sh-NC folic acid-supplemented group (N20); (6) sh-*SLC7A11* folic acid-supplemented group (S20). The stable sh-*SLC7A11* HT-22 cells and sh-NC HT-22 cells lines were incubated for 96 h with folic acid (0, 10, 20 μmol/L). (**A**) Quantitative analysis of GSH levels (n = 6/group). (**B**) Quantitative analysis of MDA levels (n = 6/group). (**C**) Quantitative analysis of cystine levels (n = 6/group). (**D**,**E**) Representative micrographs and quantitative analysis of intracellular Fe^2+^ (n = 3/group). Scale bar = 50 μm. (**F**,**G**) Representative micrographs and quantitative analysis of intracellular ROS (n = 3/group). Scale bar = 50 μm. (**H**) Representative Western blots of SLC7A11, GPX_4_, FTH1, and β-actin (n = 3/group). (**I**–**K**) The protein expression levels of SLC7A11, GPX_4_, and FTH1 were normalized to β-actin (n = 3/group). Panel (**I**) depicts SLC7A11, panel (**J**) depicts GPX_4_, and panel (**K**) depicts FTH1. Data are expressed as mean ± SD. Statistical significance is indicated as * *p* < 0.05 compared with the sh-NC folic acid-normal group; # *p* < 0.05 compared with the sh-SLC7A11 folic acid-normal group; and & *p* < 0.05 compared with the same folic acid concentration.

To investigate the potential site of folic acid’s inhibitory action on ferroptosis, *SLC7A11* expression was knocked down. Following the knockdown of *SLC7A11* gene expression, the up-regulation of GSH levels and cystine levels or the down-regulation of MDA levels by folic acid were diminished, with no significant differences observed among the various concentrations of folic acid (*p* > 0.05, Figure 5A–C). Cells with *SLC7A11* gene knockdown exhibited significant increase in Fe^2+^ and ROS levels, while the inhibitory effects of folic acid on Fe^2+^ and ROS were also reduced. Compared to the folic acid 10 μmol/L group, the levels of Fe^2+^ and ROS in the 0 μmol/L were higher (*p* < 0.05, Figure 5D–G). However, no significant difference was noted between the 10 μmol/L and 20 μmol/L groups (*p* > 0.05, Figure 5D–G). These findings suggested that the inhibition of neuronal ferroptosis, the enhancement of antioxidant capacity, and intracellular cystine levels by folic acid were compromised by the down-regulation of *SLC7A11*.

The knockdown of *SLC7A11* diminished folic acid’s ability to up-regulate the antioxidant pathway associated with ferroptosis, specifically the SLC7A11-GSH-GPX_4_ axis, and limited its up-regulation of the ferroptosis inhibitory protein FTH1. In HT-22 cells with *SLC7A11* knockdown, the expression levels of ferroptosis inhibitory proteins SLC7A11, GPX_4_, and FTH1 were significantly reduced, particularly under conditions of folic acid deficiency. Notably, within the *SLC7A11* knockdown groups, compared to the folic acid 10 μmol/L group, increasing doses of folic acid did not lead to significant changes in SLC7A11 and GPX_4_ protein levels in 20 μmol/L folic acid (*p* > 0.05, Figure 5H–J). However, FTH1 expression in the 20 μmol/L group significantly exceeded that of the 10 μmol/L groups (*p* < 0.05, Figure 5K).

These findings suggest that folic acid inhibits ferroptosis by activating the SLC7A11-GSH-GPX_4_ pathway through its interaction with SLC7A11.

## 3. Discussion

Our study demonstrated that folic acid exerted neuroprotection against age-related neuronal damage through inhibited ferroptosis. Specifically, folic acid promoted the activation of the SLC7A11-GSH-GPX_4_ antioxidant pathway. Folic acid supplementation up-regulated SLC7A11 expression, then restored GSH biosynthesis and antioxidant enzyme activity of GPX_4_, thereby attenuating brain tissue and intraneuronal iron overload, the accumulation of free radicals, and toxic lipid peroxidation. Moreover, folic acid increased cystine levels in vivo and in vitro, which may help SLC7A11 to take up cystine across membranes and act as a reaction substrate to further up-regulate GSH-dependent GPX_4_ expression. These effects were specifically dependent on SLC7A11, as genetic knockdown abolished folic acid-mediated increased in GPX_4_ activity, led to ferroptosis characteristics re-emerge accompanied by GSH depletion and cystine intake deficiency. But the knockdown of *SLC7A11* had little effect on the role of folic acid in promoting FHT1 expression. These findings established a novel link between folic acid metabolism and ferroptosis regulation, providing a potential dietary intervention for aging-related neurodegenerative disorders.

Our previous research, utilizing the same batch of rats, showed that inhibitory effect of lifelong folic acid supplementation on oxidative stress-induced neuronal apoptosis in aged rats with alleviated telomere attrition as the main mechanism [7]. In order to explore another aspect of the neuroprotective role played by folic acid, this study specifically explored the impact of folic acid in activating an important antioxidant pathway and increasing cystine uptake, ameliorating the adverse phenotype of ferroptosis, and thereby exerting attenuating effects on neuronal damage in the aging rat brain. Ferroptosis significantly contributes to neuronal damage and has been shown to be one of the co-morbid mechanisms of age-related neurodegenerative diseases [12]. Thus it is necessary to systematically discuss the possible anti-ferroptosis mechanisms of folic acid.

SLC7A11-GSH-GPX_4_ antioxidant pathway regulates ferroptosis development, which limits lipid peroxidation [13]. SLC7A11, a key subunit of the cystine/glutamate antiporter system (System Xc-), important upstream regulators of inhibiting ferroptosis [14]. The results of the present study indicated that folic acid was able to enhance the inhibitory effect of the SLC7A11-GSH-GPX_4_ antioxidant pathway on the phenomenon of ferroptosis (iron, ROS, lipid peroxide deposition) by up-regulating the expression of SLC7A11. Our molecular docking analysis further predicted the specific site of folic acid’s action, indicating that folic acid more likely acted on SLC7A11, compared to the downstream GPX_4_. Further binding affinity stability and kinetic evaluation, the active conformation of SLC7A11 was stabilized by the adaptive binding of 5-MTHF to specific amino acid residues around the binding pocket, forming multiple hydrogen bonds and a salt bridge [15]. Based on the concepts related to heterogeneous effects, we surmised that the mutual effect might induce allosteric effects, enhancing the binding or releasing of the substrate to SLC7A11, and promote the transport activity and efficiency of System Xc- [16,17]. Cystine is a transport substrate for SLC7A11; it is reduced to cysteine for GSH synthesis [18]. In in vitro cell cultures or tissue homogenates, cystine is more stable relative to cysteine and is suitable for reflecting SLC7A11 function and intracellular cystine availability [19]. This study provided strong evidence that folic acid up-regulated intracellular cystine levels, GSH synthesis, and GPX_4_ antioxidant activity in brain tissues and cellular ferroptosis models in aged rats, reflecting a positive structural and functional effect of folic acid on SLC7A11. *SLC7A11* knockdown reduced cystine transport into cells and hindered intracellular cystine and GSH metabolism, and increased lipid ROS levels in gastric cancer cells, which is consistent with our study [20]. Additionally, folic acid lowered homocysteine (Hcy) levels, alleviating oxidative stress and lipid peroxidation, which may indirectly inhibit ferroptosis [7,21].

Folic acid acts as a methyl donor and regulates DNA synthesis and methylation, influencing gene expression through this epigenetic modification [22]. In an Alzheimer’s disease (AD) model, folic acid protected neuronal cells from DNA methylation alterations of the APP and PS1 genes by amyloid β oligomers and attenuated neuronal toxicity [23]. Our findings demonstrated that folic acid supplementation produces more significant effects in untreated cells compared to Erastin-treated cells, suggesting its cellular impact extends beyond ferroptosis regulation. In metabolically stable neurons, folic acid markedly increased GSH levels while reducing MDA and LPO accumulation (all *p* <0.05), and up-regulated expression of SLC7A11, GPX4, FTH1, and cystine, highlighting its dual role as both a methyl donor and antioxidant in healthy cellular environments. While folic acid showed minimal effects on ferroptosis markers (Fe^2+^ and ROS) in untreated cells, it significantly enhanced existing antioxidant defense systems. In our experimental results, folic acid was found to have a functional improving effect on ferroptosis neurons, but the antioxidant effect was not as pronounced as in cells under normal conditions. Its attenuated effects in ferroptosis cells likely result from irreversible antioxidant system collapse and structural damage that surpass critical recovery thresholds. These differential responses underscore that folic acid’s neuroprotective efficacy depends fundamentally on cellular metabolic integrity. Although our study focused on ferroptosis, these observations reveal broader cellular functions of folic acid that merit further exploration, particularly concerning its dose–response relationships across physiological and pathological states.

The role of folic acid in enhancing the expression of SLC7A11 may be related to more upstream regulators. Under oxidative stress conditions, folic acid can up-regulate the protein expression of Silent information regulator sirtuin 1 (SIRT1), activate Nuclear factor erythroid 2-related factor 2 (Nrf2), and initiate the transcriptional expression of multiple downstream antioxidant target genes, thereby enhancing cellular resistance to oxidative stress and exogenous toxic substances [24,25]. Studies have demonstrated that folic acid (3 µg/kg) can activate Nrf2 in rats with acute kidney injury [26], while *Nrf2* gene knockout abolished the protective effects of folic acid against oxidative stress in melanocytes [27]. Furthermore, *Nrf2* promotes *SLC7A11* transcription by binding to the antioxidant response element (ARE) in the *SLC7A11* promoter region [28]. It is well known that there is a close and mutually reinforcing relationship between oxidative stress and ferroptosis. These findings combined with the results of our experiments suggest that when ferroptosis occurs, folic acid may regulate SLC7A11 expression through the Nrf2 pathway. In future studies, we plan to further investigate the relationship between folic acid and upstream regulators of SLC7A11 such as Nrf2 and SIRT1, aiming to elucidate the precise mechanisms by which folic acid enhances SLC7A11 expression. This research may provide new therapeutic targets for mitigating ferroptosis-related neuronal damage.

FTH1 modulates iron metabolism by converting Fe^2+^ to Fe^3+^ to prevent iron accumulation and ROS generation [29]. One of the manifestations of overall organismal functional aging is iron absorption dysfunction, where excess iron accumulation makes senescent tissue cells more susceptible to oxidative stress and triggers iron-dependent cell death [30]. Folic acid regulated intracellular iron concentrations and enhanced GPX_4_ expression in mouse neurons and nucleus pulposus cells [21,31]. Notably, this study showed that the effect of folic acid on SLC7A11 existed independently of FTH1. Overall, folic acid also improved iron metabolism and the expression of related proteins and inhibited the undesirable phenomenon of ferroptosis.

While most studies focus on folic acid’s role in non-neuronal ferroptosis (e.g., renal injury or cancer [32]), our work uniquely demonstrated its neuroprotective efficacy in aging models. Notably, high-dose folic acid (250 mg/kg) can paradoxically induce ferroptosis in kidneys [33], underscoring the importance of dose and tissue specificity in folic acid’s effects.

## 4. Materials and Methods

### 4.1. Animals and Dietary Treatment

All rats were obtained from the Beijing Vital River Laboratory Animal Technology Co., Ltd., Beijing, China. All experimental procedures were approved by the Animal Ethics Committee of Tianjin Medical University (approval number: TMUaMEC2019003; 24 April 2019), and conformed to the Guide for the Care and Use of Laboratory Animals published by the National Institutes of Health. The study design had been detailed in a published document [7] and was summarized as follows. Three-month-old male Sprague Dawley (SD) rats were randomly assigned to one of three intervention groups (30 rats in each group) using a computer-generated random number table: (1) the folic acid-deficient diet group (FA-D), which received a diet containing less than 0.1 mg/kg folic acid; (2) the folic acid-normal diet group (FA-N), which received a diet of 2.0 mg/kg; (3) the folic acid-supplemented diet group (FA-S), which received a diet of 4.0 mg/kg. Dietary intervention was carried out by providing the respective diets ad libitum for 22 months, starting from the age of 3 months until 25 months. At 14, 21, and 25 months of age, 10 rats in each group were sacrificed for analysis [7]. In this study, only 25-month-old rats were used for analysis. In addition, a group of four-month-old male SD rats served as the young control (Con-Y) group.

### 4.2. Cell Culture and Treatment

Mouse HT-22 neuronal cells were procured from Shanghai Cell Bank at the Chinese Academy of Military Medical Sciences. Cells were cultured in high-glucose Dulbecco’s Modified Eagle Medium (DMEM, BasalMedia, L130KJ, Shanghai, China) supplemented with 10% fetal bovine serum (FBS, Gibco, A5256701, Carlsbad, CA, USA) and 1% penicillin–streptomycin solution (Gibco, 15070063, Carlsbad, CA, USA), maintained at 37 °C in a humidified incubator with an atmosphere of 5% CO_2_, and passaged using 0.25% trypsin–EDTA (Gibco, 25200056, Carlsbad, CA, USA) when they reached 90% confluence. For experimental purposes, HT-22 cells were incubated for 72 h with various concentrations of folic acid (ranging from 0 to 20 μmol/L) and then incubated for 24 h with a combination of folic acid and either Erastin (10 μmol/L) or vehicle. Folic acid-free DMEM powder (BasalMedia, customization, Shanghai, China) was reconstituted according to the manufacturer’s instructions. Subsequently, appropriate amounts of folic acid (Solarbio, No. F8090, Beijing, China) were dissolved in dimethyl sulfoxide (DMSO) and added to the reconstituted DMEM medium to prepare culture media with the desired folic acid concentrations for the experiments.

### 4.3. Sh-SLC7A11 Lentivirus Transfection and Cell Treatment

To down-regulate *SLC7A11* in HT-22 cells, the cells were seeded in 6-well plates at a density of 2.5 × 10^5^/mL and cultured at 37 °C with 5% CO_2_, following the manufacturer’s guidelines. Cells were transfected with lentiviral vectors (sh-*SLC7A11* #1-3 or sh-NC) at 2 μg/well using polybrene (8 μg/mL, Sigma, TR-1003-G, J, Shanghai, China) to enhance infection efficiency. Lentiviral vectors were purchased from BrainVTA (Wuhan, China). The confirmation of successful transfection was achieved by monitoring green fluorescence at 48 h and performing real-time PCR. Off-target rates were predicted using TargetScan Human 8.0 and validated by RNA sequencing. The sh-*SLC7A11* #3, which exhibited the lowest off-target rate of 28.8%, was selected for constructing the stable knockdown cell line, alongside sh-NC. Lentivirus transfection was conducted at a multiplicity of infection (MOI) of 20, and MOI value = virus titer (TU/mL) × virus volume (mL)/cell number. Puromycin selection (2 μg/mL) was maintained for 7 days, with medium changes every 2 days. Fluorescence was monitored daily using a fluorescence microscope (Nikon Eclipse Ti, Shanghai, China) until ≥ 98% of cells exhibited GFP expression. The shRNA sequences were as follows: sh-*SLC7A11* #3 (LV-2752), forward: 5′-GCCCTGTCCTATGCAGAATTA-3′; reverse: 5′-TAATTCTGCATAGGACAGGGCT-3′ (none in human and mouse).

Cell culture conditions were detailed in 2.2. For experimental purposes, the stable sh-*SLC7A11* HT-22 cells and sh-NC cell lines were incubated for 96 h with various concentrations of folic acid (ranging from 0 to 20 μmol/L).

### 4.4. DAB-Enhanced Perls’ Staining

Fe^3+^ deposition in 5 μm brain sections was detected using a 3,3’-diaminobenzidine (DAB)-enhanced Perls’ staining kit (Solarbio, G1428, Beijing, China). As instructed, sections were incubated with Perls’ staining solutions. Fe^3+^ positive staining (brown dots) was quantified using Image-Pro Plus Version 6.0 image analysis software to measure integrated optical density (IOD).

### 4.5. Brain Tissue Fe^2+^ Assay

Fe^2+^ concentrations in brain tissue of rats were measured using a ferrous content detection kit (AIDISHENG, ADS-F-QT027, Jiangsu, China). Rat brain tissue homogenate was centrifuged and collected the supernatant; absorbance was measured at 562 nm.

### 4.6. Cystine Level Assay

Cystine levels of rat brain tissue and cells (5 × 10^5^/well) in 6-well plates were measured using cystine ELISA kits (YaJi Biological, YS08852B, YS16037B, Shanghai, China) according to the manufacturer’s instructions. Total proteins were measured using a BCA protein assay kit (SparkJade, EC0001-A, Shandong, China).

### 4.7. Immunohistochemical Staining

The rabbit two-step detection kit (Beijing Zhongshan Golden Bridge Biotechnology Co., Ltd., PV-9001, Beijing, China) was used. The 5 μm brain sections were dewaxed, rehydrated, rinsed and antigen-repaired with citrate (pH 6.0) or Tris/EDTA buffer (pH 9.0). After blocking, the sections were incubated with the following primary antibodies overnight at 4 °C: SLC7A11 (1:800, abcam (Shanghai), ab307601, Shanghai, China), GPX_4_ (1:800, proteintech, 30388-1-AP, Wuhan, China), FTH1 (1:1000, Signalway, Antibody, #49644, College Park, MD, USA). Enhancer and enzyme-labeled goat anti-rabbit IgG polymer were applied, followed by DAB staining. The IOD was measured by Image-Pro Plus Version 6.0 image analysis software.

### 4.8. Western Blot

Rats’ brain tissue homogenate or HT-22 cells were lysed with radio immunoprecipitation assay (RIPA), and protein concentrations were measured using a BCA kit (Spark Jade, EC0001, Shandong, China). Protein concentrations were adjusted with loading buffer, and 20 μg of protein was loaded per well. The electrophoresis conditions were 80 V, 30 min to 100 V 60 min, and the electrotransfer conditions were 200 mA, 90 min. PVDF membranes were blocked with 5% skimmed milk, incubated overnight at 4 °C with specific primary antibodies: (1:2000, Abmart, P30002, Shanghai, China), SLC7A11 (1:1000, T57046, Abmart, Shanghai, China), GPX_4_ (1:2000, T56959, Abmart, Shanghai, China), FTH1 (1:1000, T55648F, Abmart, Shanghai, China). Membranes were incubated with Goat Anti-Rabbit IgG H&L antibody (1:10000, bs-0295G-HRP, Bioss, Beijing, China) for 1 h. They were then developed using enhanced chemiluminescence (ECL) and photographed. Proteins and bands were analyzed by Image-Pro Plus Version 6.0 image analysis software.

### 4.9. Cell Viability Assay

Cell viability was assessed using an enhanced CCK-8 kit (Spark Jade, CT0001, Shandong, China). HT-22 cells (5 × 10^3^/well) were seeded in 96-well plates, incubated with CCK-8 solution for 3 h, absorbance values of each measuring hole were read at 450 nm of the microplate reader.

### 4.10. Intracellular Fe^2+^, LPO, and ROS Assays

For Fe^2+^ detection, cells were stained with 3 μmol/L FerroOrange (Meilun Biological, MA0647, Liaoning, China). For LPO, cells were stained with 5 μmol/L C11 BODIPY 581/591 (GLPBIO, GC40165, Montclair, CA, USA). For ROS, Erastin-induced ferroptosis cells were stained with 10 μmol/L green fluorescent probe DCFH-DA (Beyotime, S0033S, Shanghai, China), while sh-*SLC7A11* or sh-NC cells were stained with 10 μmol/L red fluorescent probe DHE (Solarbio, CA1420, Beijing, China), because lentivirus exhibits green fluorescence. Then, co-treatment with Hoechst 33342 was performed. After 30 min incubation at 37 °C, the cells were observed under a fluorescence inverted microscope. The fluorescence intensity was measured by Image-Pro Plus Version 6.0 image analysis software.

### 4.11. MDA and GSH Assays

Cells (5 × 10^5^/well) in 6-well plates, MDA (Nanjing Jiancheng, A003-1, Nanjing, China), and GSH (Nanjing Jiancheng, A006-2-1, Nanjing, China) levels were measured using respective kits. Total proteins were measured using a BCA protein assay kit.

### 4.12. Molecular Docking

The structure of 5-MTHF was prepared by Chem Draw software v23.1.1 (PerkinElmer, Waltham, MA, USA). The crystal structure of SLC7A11 protein was predicted by AlphaFold2, and the crystal structure of GPX_4_ protein was obtained in Protein Data Bank (PDB ID:5L71). Proteins and 5-MTHF structures were prepared in Schrödinger. XP GScore and MM-GBSA dG Bind were used in molecular docking to evaluate the stability of ligand–protein binding [34,35]. Lower XP GScore or MM-GBSA dG Bind value indicated higher binding stability of 5-MTHF to the protein, with XP GScore typically < −6 and MM-GBSA dG Bind < −30 kcal/mol.

### 4.13. Statistical Analysis

The data were expressed as mean ± SD. Comparisons among the different groups were performed by one-way ANOVA in vivo, and by two-way ANOVA in vitro, followed by LSD-t test for multiple comparisons. SPSS 24.0 (SPSS, Chicago, IL, USA)was used for statistical analysis, which were considered statistically significant at *p* < 0.05. GraphPad Prism 9.3.0 (GraphPad Software, La Jolla, CA, USA) was used to draw statistical graphics.

## 5. Conclusions

In conclusion, folic acid supplementation is shown to effectively inhibit neuronal ferroptosis and alleviate aging-related neuronal damage both in vivo and in vitro. The underlying mechanism might involve the crucial interaction of folic acid with SLC7A11, which up-regulated SLC7A11-GSH-GPX_4_ antioxidant pathway. Furthermore, folic acid improved cystine levels to enhance the production of GSH, elevated antioxidant capacity, and reduced lipid peroxidation in neuronal cells. The study established that aging can induce neuronal cells oxidative damage through ferroptosis. These findings suggested that folic acid supplementation could be a novel therapeutic strategy to combat ferroptosis in age-related neuronal damage.

## Data Availability

The datasets and materials used and/or analyzed during the current study are available from the corresponding author on reasonable request.

## Abbreviations

The following abbreviations are used in this manuscript:

AD	Alzheimer’s disease
DAB	3,3’-diaminobenzidine
DMEM	Dulbecco’s Modified Eagle Medium
DMSO	Dimethyl sulfoxide
ECL	Enhanced chemiluminescence
FBS	Fetal bovine serum
FTH1	Ferritin heavy chain 1
GPX_4_	Glutathione peroxidase 4
GSH	Glutathione
GScore	GlideScore
Hcy	Homocysteine
HHcy	Hyperhomocysteinemia
IOD	Integrated optical density
LPO	Lipid peroxidation
MDA	Malondialdehyde
MM-GBSA dG Bind	Molecular mechanics/generalized born surface area f binding free energy
MOI	Multiplicity of infection
NRF2	Nuclear factor erythroid 2-related factor 2
PDB	Protein data bank
RIPA	Radio immunoprecipitation assay
ROS	Reactive oxygen species
SD	Sprague Dawley
SIRT1	Silent information regulator sirtuin 1
SLC7A11	Solute carrier family 7 member 11
System Xc-	Cystine/glutamate antiporter system
XP	Extra precision
5-MTHF	5-methyltetrahydrofolate

## References

[1] Harper S. (2014). Economic and Social Implications of Aging Societies. Science.

[2] Bloom D., Chatterji S., Kowal P., Lloyd-Sherlock P., Mckee M., Rechel B., Rosenberg L., Smith J.P. (2015). Macroeconomic Implications of Population Ageing and Selected Policy Responses. Lancet.

[3] Mavaddatiyan L., Naeini S., Khodabandeh S., Hosseini F., Skelton R.P., Azizi V., Talkhabi M. (2025). Exploring the Association between Aging, Ferroptosis, and Common Age-Related Diseases. Arch. Gerontol. Geriatr..

[4] Xu L., Liu Y., Chen X., Zhong H., Wang Y. (2023). Ferroptosis in Life: To Be or Not to Be. Biomed. Pharmacother..

[5] Trushina E., McMurray C.T. (2007). Oxidative Stress and Mitochondrial Dysfunction in Neurodegenerative Diseases. Neuroscience.

[6] Gleitze S., Paula-Lima A., Núñez M.T., Hidalgo C. (2021). The Calcium-Iron Connection in Ferroptosis-Mediated Neuronal Death. Free Radic. Biol. Med..

[7] Zhou D., Sun Y., Dong C., Wang Z., Zhao J., Li Z., Huang G., Li W. (2024). Folic Acid Alleviated Oxidative Stress-Induced Telomere Attrition and Inhibited Apoptosis of Neurocytes in Old Rats. Eur. J. Nutr..

[8] Chen T.-F., Tang M.-C., Chou C.-H., Chiu M.-J., Huang R.-F.S. (2013). Dose-Dependent Folic Acid and Memantine Treatments Promote Synergistic or Additive Protection against aβ(25–35) Peptide-Induced Apoptosis in SH-SY5Y Cells Mediated by Mitochondria Stress-Associated Death Signals. Food Chem. Toxicol..

[9] Sonkar S.K., Kumar S., Singh N.K., Tandon R. (2021). Hyperhomocysteinemia Induced Locked-in Syndrome in a Young Adult Due to Folic Acid Deficiency. Nutr. Neurosci..

[10] Wu J., Liu Q., Zhang X., Wu X., Zhao Y., Ren J. (2021). STING-Dependent Induction of Lipid Peroxidation Mediates Intestinal Ischemia-Reperfusion Injury. Free Radic. Biol. Med..

[11] Liu L., Liu R., Liu Y., Li G., Chen Q., Liu X., Ma S. (2021). Cystine-Glutamate Antiporter xCT as a Therapeutic Target for Cancer. Cell Biochem. Funct..

[12] Zhou R.-P., Chen Y., Wei X., Yu B., Xiong Z.-G., Lu C., Hu W. (2020). Novel Insights into Ferroptosis: Implications for Age-Related Diseases. Theranostics.

[13] Ming T., Lei J., Peng Y., Wang M., Liang Y., Tang S., Tao Q., Wang M., Tang X., He Z. (2024). Curcumin Suppresses Colorectal Cancer by Induction of Ferroptosis via Regulation of P53 and Solute Carrier Family 7 Member 11/Glutathione/Glutathione Peroxidase 4 Signaling Axis. Phytother. Res..

[14] Yan R., Xie E., Li Y., Li J., Zhang Y., Chi X., Hu X., Xu L., Hou T., Stockwell B.R. (2022). The Structure of Erastin-Bound xCT-4F2hc Complex Reveals Molecular Mechanisms Underlying Erastin-Induced Ferroptosis. Cell Res..

[15] Stank A., Kokh D.B., Fuller J.C., Wade R.C. (2016). Protein Binding Pocket Dynamics. Acc. Chem. Res..

[16] Gose T., Shafi T., Fukuda Y., Das S., Wang Y., Allcock A., Gavan McHarg A., Lynch J., Chen T., Tamai I. (2020). ABCG2 Requires a Single Aromatic Amino Acid to “Clamp” Substrates and Inhibitors into the Binding Pocket. FASEB J..

[17] Cortés-Hernández P., Domínguez-Ramírez L. (2017). Role of Cis-Trans Proline Isomerization in the Function of Pathogenic Enterobacterial Periplasmic Binding Proteins. PLoS ONE.

[18] Matsumura N., Kinoshita C., Aoyama K. (2021). Mechanism of glutathione production in neurons. Nihon Yakurigaku Zasshi.

[19] Alvarez E., Ramón F., Magán C., Díez E. (2004). L-cystine inhibits aspartate-beta-semialdehyde dehydrogenase by covalently binding to the essential 135Cys of the enzyme. Biochim. Biophys. Acta.

[20] Feng H., Yu J., Xu Z., Sang Q., Li F., Chen M., Chen Y., Yu B., Zhu N., Xia J. (2024). SLC7A9 Suppression Increases Chemosensitivity by Inducing Ferroptosis via the Inhibition of Cystine Transport in Gastric Cancer. eBioMedicine.

[21] Zhang X., Huang Z., Xie Z., Chen Y., Zheng Z., Wei X., Huang B., Shan Z., Liu J., Fan S. (2020). Homocysteine Induces Oxidative Stress and Ferroptosis of Nucleus Pulposus via Enhancing Methylation of GPX4. Free Radic. Biol. Med..

[22] Cui S., Lv X., Li W., Li Z., Liu H., Gao Y., Huang G. (2018). Folic acid modulates VPO1 DNA methylation levels and alleviates oxidative stress-induced apoptosis in vivo and in vitro. Redox Biol..

[23] Liu H., Li W., Zhao S., Zhang X., Zhang M., Xiao Y., Wilson J.X., Huang G. (2016). Folic Acid Attenuates the Effects of Amyloid β Oligomers on DNA Methylation in Neuronal Cells. Eur. J. Nutr..

[24] Chen B., Lu Y., Chen Y., Cheng J. (2015). The Role of Nrf2 in Oxidative Stress-Induced Endothelial Injuries. J. Endocrinol..

[25] Zhang H., Zhang X., Wang Y., Zhao X., Zhang L., Li J., Zhang Y., Wang P., Liang H. (2023). Dietary folic acid supplementation attenuates maternal high-fat diet-induced fetal intrauterine growth retarded via ameliorating placental inflammation and oxidative stress in rats. Nutrients.

[26] Wijerathne C.U., Au-Yeung K., Siow Y., O K. (2022). 5-methyltetrahydrofolate attenuates oxidative stress and improves kidney function in acute kidney injury through activation of Nrf2 and antioxidant defense. Antioxidants.

[27] Du P., Zhang S., Li S., Yang Y., Kang P., Chen J., Gao T., Li C., Zhang Q., Zhang W. (2021). Folic Acid Protects Melanocytes from Oxidative Stress via Activation of Nrf2 and Inhibition of HMGB1. Oxidative Med. Cell. Longev..

[28] Dai E., Chen X., Linkermann A., Jiang X., Kang R., Kagan V.E., Bayir H., Yang W.S., Garcia-Saez A.J., Ioannou M.S. (2024). A Guideline on the Molecular Ecosystem Regulating Ferroptosis. Nat. Cell Biol..

[29] Zhu L., Bao Y., Liu Z., Liu J., Li Z., Sun X., Zhou A., Wu H. (2024). Gualou-Xiebai Herb Pair Ameliorate Atherosclerosis in HFD-Induced ApoE-/- Mice and Inhibit the Ox-LDL-Induced Injury of HUVECs by Regulating the Nrf2-Mediated Ferroptosis. J. Ethnopharmacol..

[30] Zhao T., Guo X., Sun Y. (2021). Iron Accumulation and Lipid Peroxidation in the Aging Retina: Implication of Ferroptosis in Age-Related Macular Degeneration. Aging Dis..

[31] Zhang Q., Wu H., Zou M., Li L., Li Q., Sun C., Xia W., Cao Y., Wu L. (2019). Folic Acid Improves Abnormal Behavior via Mitigation of Oxidative Stress, Inflammation, and Ferroptosis in the BTBR T+ Tf/J Mouse Model of Autism. J. Nutr. Biochem..

[32] Zhang D., Zhang M., Pang Y., Li M., Ma W. (2024). Folic Acid-Modified Long-Circulating Liposomes Loaded with Sulfasalazine for Targeted Induction of Ferroptosis in Melanoma. ACS Biomater. Sci. Eng..

[33] Martín-Sánchez D., Ruiz-Andrés O., Poveda J., Carrasco S., Cannata-Ortiz P., Sanchez-Niño M.D., Ortega M.R., Egido J., Linkermann A., Ortiz A. (2017). Ferroptosis, but Not Necroptosis, Is Important in Nephrotoxic Folic Acid-Induced AKI. J. Am. Soc. Nephrol. JASN.

[34] Wang W., Di T., Wang W., Jiang H. (2023). EGCG, GCG, TFDG, or TSA Inhibiting Melanin Synthesis by Downregulating MC1R Expression. IJMS.

[35] Dong X.-N., Li M.-T. (2024). Inhibitory Effect of Aloperine on Transient Outward Potassium Currents in Rat Cardiac Myocytes. Front. Pharmacol..

